# Household ICT Utilization and Food Security Nexus in Nigeria

**DOI:** 10.1155/2021/5551363

**Published:** 2021-11-10

**Authors:** Jeremiah O. Ejemeyovwi, Romanus Osabohien, Bosede Ngozi Adeleye, Tyrone De Alwis

**Affiliations:** ^1^Department of Economics and Development Studies & Centre for Economic Policy and Development Research-CEPDeR, Covenant University, Ota, Nigeria; ^2^Department of Economics, University of Colombo, Sri Lanka

## Abstract

The study examines the nexus between household ICT utilization and food security in Nigeria, which supports goal 2 of the 2030 Sustainable Development Goals (SDGs) that aims to “*end hunger, achieve food security, improved nutrition, and promote sustainable agriculture.*” The study employs the logit regression to wave 4 of Living Standard Measurement Integrated Survey on Agriculture (LSMS) data for the empirical analysis. Based on the analysis, the study finds that for male household, ICT utilization has a statistically significant and positive nexus with food security. In contrast, for the female households, an insignificant and, however, negative nexus is observed with food security in Nigeria. Furthermore, the findings show that for male household users, a 1 percent increase in male household ICT utilization spurs about 0.68 percent increase in food security in Nigeria. The findings imply that among the male and female household ICT users, the male household ICT utilization is significantly contributes to food security in Nigeria. The study recommends that relevant stakeholders take strategic measures to ensure that the potentials of household ICTs be fully maximized to contribute to food security in the nearest future as confirmed by studies in other regions.

## 1. Introduction

Food security according to the United Nations [1] refers to a state of wellbeing whereby the citizens of an economy have physical, social, and economic access to adequate, safe, and nutritious food which meets the food requirements and also dietary needs to gain active and healthy life. Food can have a significant impact on the human body, and hence the term “*food securit*y” has become a part and parcel of all aspects of life (Food and Agricultural Organisation [2, 3]). According to the World Economic Forum [4], 108 million around the world are suffering from the crisis known as “food security” as around 50 per cent eat food that is undernutritious, and as a result, one out of nine suffers from both hunger and malnutrition [4]. However, food security can be improved by managing crop activities through building sustainable food security and agricultural practices. Undeniably, agriculture has been established to be a major source of livelihood in Nigeria [5, 6]. It is a means of livelihood for about 70% of the total labor force, accounting for about two-fifth of Nigeria's gross domestic product-GDP [7]. Given the level of involvement of majority of the labor force, food security is expected to not be an issue for and country like Nigeria but on the contrary, for many African countries, Nigeria inclusive, there is high prevalence of undernutrition and food insecurity at both the national and household levels [5].

Despite the relatively impressive performance of the agriculture sector in Nigeria, the issue of food insecurity subsists. Food and Agriculture Organization (FAO) data on food security (proxied by average value of food production) when compared with high-income countries and upper-middle income countries show that as on 2011-2013, Nigeria reported $198 per caput while high-income countries and upper middle-income countries reported $491 per caput and $372 per caput, respectively. More recently, as on 2015-2017, Nigeria reported $137 per caput while high-income countries and upper middle-income countries reported $336 per caput and 257 per caput (Food and Agriculture Organization–FAO [8]. This implies that despite the large contribution of the agriculture to the labor sector and to the country's GDP, there is still the issue of relative food insecurity in Nigeria; thus, the challenges of food security need to be addressed.

Common challenges of food security include harsh agroecology characterized by low soil fertility, recurrent droughts and/or floods, and unpredictable weather patterns associated with climate change [7]. Farmers who witness these challenges tend to be vulnerable, but also faced technological deficits. These technological deficits include roads and transport networks, telecommunications, potable water, and irrigation. The aftermath is low production and reduced supply of food, which in turn translates to food insecurity. Furthermore, other challenges to food security include poor infrastructure, low access to credit, ineffective policies, weak institutional framework (lack of accountability in agricultural governance), lack of relevant and accurate information on production practices, poor farm management, high prices of agricultural produce, inadequate food security dimensions, and markets for agricultural products that can better the lots of farmers [9, 10].

A potential solution for curbing the challenges food insecurity among rural households in developing countries is knowledge building and information sharing [11]. Information and communication technology (ICT) becomes handy in addressing most of the challenges of food insecurity through knowledge building and real time as well as automated forecasted information sharing [9]. ICTs are comprised of various collections of resources and technical tools used for connecting, spreading, storing, and managing information [12, 13]. ICT utilization impacts food security by improving communication among research systems and farmers and also improving accessibility to information regarding inputs and technologies [14]. It provides more rapid accessibility to high quality information such as weather forecasts for reduced weather unpredictability and ensuring information dissemination at appropriate times and places for sales of agricultural products, increasing agricultural products, and decreasing agricultural product losses [15].

Theoretically, the impact of ICT on food security is unarguable but for a country like Nigeria, the current high level of utilization and agriculture disposition supports the need to empirically investigate the impact of ICT on food security. Studies in literature attempted an empirical discourse about the role of mobile technology on food security in Nigeria [5, 16] and in other African countries [17–19]. Olaniyi and Ismaila [5] and Yusuff, Majeldunmi, Adedeji, and Adams [16] established that majority of the communication technologies used by farmers are radio and mobile phones, but however, to the best of knowledge, the relationship between ICT and food security in Nigeria is yet to be empirically investigated. For instance, Namubiru, Ngaka, and Picho [20] assess the nexus between ICT and food security in Uganda and the study found that ICT improves food security status by about 38 percent while Sakata [19] positioned an inquiry on the relevance of ICT as the basis of precision agriculture in Vietnam. Thus, from the scarce literature on ICT-food security nexus in Nigeria, the specific role on ICT has not received the attention it deserves.

The discussed lacuna in the literature creates opportunities for improvement in some areas: first, contrary to engaged regional panel studies that are characterized by policy implications of broad scope and the few existing country-based studies, it is important to utilize a robust survey data that truly captures the reflections of the people as against the trend of using country wide aggregated data for more specific and uniquely tailored policy outcomes [7] and second, some studies argue that food security springs up from equal distribution of resources and information [18]. Given these, attempt is made to tests these hypotheses for Nigeria by building on the studies of Namubiru, Ngaka, and Picho [20] as well as Yusuff, Majekodunmi, Adedeji, and Adams [16]. Third, the study further disaggregates ICT utilization into both the male and female household ICT utilization to add to knowledge the comparative impacts on food security in Nigeria.

### 1.1. Literature Review

Over the past few decades, ICT played an important role in Africa's development process. However, in incorporating ICT to a sector like agriculture, which contributes immensely to the economic development in Africa, clearly defined ideas are required. Scholars have attempted to investigate how ICT can be adopted in order to ensure food security in Africa due to economic advantages, which include opportunities for economic growth and poverty alleviation. Chavula [21] studied the role of ICT in agricultural production in Africa from 2000 to 2011 using panel data for 34 African countries. The study found that the ICT plays a significant role in enhancing agricultural production. However, the study further concluded that mobile phones are having relatively fewer roles; yet, main lines contribute significantly to agricultural growth.

Likewise, Namubiru, Ngaka, and Picho [18] examined the effect of ICT on household's food security in Uganda giving special reference to Acoholi subregion. Cross-sectional and descriptive design was utilized, and the results indicated an average of 18.2% of the households in Acholi subregion used ICT for food security while 31.9% did not use ICT for food security. Although Chavula [21] found that mobile phones are having less role, Namubiru, Ngaka, and Picho [20] found completely opposite as the majority of population do have access to food through mobile phones. Correspondingly, on a study on India, Syiem & Raj [22] found that ICT was heavily used in farming. The same observation was made by Olaniyi [5] and Agwu et al. [23] in Nigeria suggesting that ICT has positive impact on agricultural production and food security. Syiem & Raj [22], Olaniyi [5], and Agwu et al. [23] hold similar views to that ICT promotes agriculture productivity.

On a related note, Aldosari et al. [17] sought to find how farmers use mobile phone technology in Saudi Arabia. Using primary data collected through questionnaires, findings revealed that 77% of the respondents used mobile phone technology, and 66% were of the view that cell phones were highly useful for their needs in relation to agriculture. The study further showed that about 66% of farmers use mobile phones to decide on the inputs of rice such as seeds, fertilizers, and pesticides. Also, Bayes [24], Gupta [25], Malhan and Rao [26], Tchouassi [27], and Arayesh [28] indicated how the usage of ICT overcame the traditional obstacles in interpersonal connection by way of increased access to information in the local markets on price and inputs. All these studies were proved previously by the Kashem [29] as cell phones reaching the 5^th^ place among all communication media especially with regards to rural farmers in providing information. Before Kashem [29], Burke and Sewake [30] maintained that the mobile internet provides significant volume of useful information related to agriculture and its associated problems. Similar study in Ghana by Overa [31] indicated that farmers could be directly connected with buyers through cell phones so that they can get a better price than working through brokers. Studies by Muto and Yamano [32], Muto and Yamano [33], and Lee and Bellemare [34] show that farmers use the mobile phone to save time, money, and energy. Ndyetabula and Legg [35] further indicated that cell phone can be used to disseminate information about the outbreak of crop diseases. However, Murthy [36] concluded that the short message services (SMS) are in order to keep the farmers regarding the weather updates.

Olaniyi [5] found that poor performance of agricultural sector led to problems in food availability and access and utilization problems in households at national levels. This was due to the fact that those involved in farming have not fully discovered likely potential of ICT for food security. Based on the above premise, the researcher assessed the ICT usage on food security, Ondo State, Nigeria. Multistage technique was applied by taking 212 farmers as a sample. It was found that cell phone, radio, and television were the highly used ICT tool for accessing information on food security. The researcher, through his empirical investigation, has recommended that institutions should concentrate on using cell phones in disseminating appropriate and timely information to farmers to ensure sustainable food security.

According to International Telecommunication Union (ITU) Telecommunication Standardization Bureau [37], “food security has become one of the main issues on the global agenda.” IITU's Telecommunication Standardization Sector (ITU-T) made efforts in this light to examine the ways in which information and communication technology (ICT) can be applied to address the problem locally and internationally by first identifying the related food security issues which could be addressed by ICT. ITU-T identified that extreme weather patterns can have impacts on availability of food; population growth caused rising demand alongside the loss of farm land due to developments. Hence, ICT is fundamental in providing the farmers with useful information regarding weather, crop prices which make them more knowledgeable about new techniques in farming. Based on the issues from the literature, this study makes efforts to examine the utilization of household ICT and its effect on food security in Nigeria with the aim to identify and address the specific issues observed in the Nigerian agricultural sector.

## 2. Materials and Methods

### 2.1. Data

This study uses wave 4 of the LSMS-ISA World Bank Survey data consisting of 7,634 households for the postplanting and postharvesting seasons. The dependent variable is food security status which is a dichotomous variable. It takes the value of 1 if the household has food security and 0; otherwise, the main explanatory variable is mobile phone usage an indicator of ICT utilization. The households are further categorized into four: if headed by male or female and if located in the urban or rural areas. Other household characteristics included in the model are educational qualification, age, marital status, state of origin, enumeration area, and sector (see Table 1). Data collected are analyzed using descriptive statistical tools such as frequency counts, percentages, means, median, and logit regression to assess the effect of ICT from the categorized households on household food security while controlling for other household characteristics. In other words, the probability of a household being food secure is analyzed using the socioeconomic characteristics of the respondents and usage of mobile phones. More details on some variables and their components are found in Table 2.

### 2.2. Model Specification

In this study, attempt is made to evaluate the impact of ICT utilization (mobile phones usage) on food security. Study adapts and follows the modeling techniques of Díaz-Pérez, Carreño-Ortega, Gómez-Galán, Callejón-Ferre [38], and Olumuyiwa and Kayode [39] and specifies the following implicit model:

(1) $$\begin{array}{c} Y_{ijk}=f\left({ICT}_{ijk},X_{ijk}\right), \end{array}$$

where *Y* is a dichotomous variable that takes the value of 1 if the household has food security and 0 otherwise; subscript *i* represents a household; *j* (*j* = 1, 2) represents the two sexes (male, female), *k* (*j* = 1, 2) represents the two sectors (urban, rural); ICT represents the utilization of ICT (mobile phone usage); *X* is the vector of household characteristics (educational qualifications, state of origin, age, hours worked, hourly wage, marital status, and enumeration area). Given that *Y*_*ijk*_ is a binary variable, Equation (1) can be rewritten as a dummy variable model and explicitly expressed as

(2) $$\begin{array}{c} Y_{ijk}=\alpha +\gamma {ICT}_{ijk}+b_{1}X_{1ijk}+\cdots +b_{n}X_{nijk}+u_{ijk}, \end{array}$$

where *E*(*u*_*ijk*_\*ICT*_*ijk*_, *X*_1*ijk*_, ⋯, *X*_*nijk*_) = 0 (for zero conditional mean assumption of ordinary least squares (OLS)). That is, the expected value of *Y*_*ijk*_ is a linear function of the regressors:

(3) $$\begin{array}{c} E\left(Y_{ijk}\right)=\alpha +\gamma {ICT}_{ijk}+b_{1}X_{1ijk}+\cdots +b_{n}X_{nijk}. \end{array}$$

The probability (*P*) that the event took place is termed “success,” and it is stated as *P*_*ijk*_ ➔ *Y*_*ijk*_ = 1(*P*_*ijk*_ = Pr(*Y*_*ijk*_ = 1) while the probability that the event did not occur is termed “failure”“and expressed as 1 − *P*_*ijk*_ ➔ *Y*_*ijk*_ = 0(1 − *P*_*ijk*_ = Pr(*Y*_*ijk*_ = 0). Hence, *Y*_*ijk*_ follows the Bernoulli probability distribution. In other words, the probability that ICT and other covariates affect food security is written as

(4) $$\begin{array}{c} P\left(Y_{ijk}=1\;|\;{ICT}_{ijk},X_{ijk}\right)=\alpha +\gamma {ICT}_{ijk}+b_{1}X_{1ijk}+\cdots +b_{n}X_{nijk} \end{array}$$

where the response probability is linear in the parameters: *γ* and *b*_*n*_ and measures the change in the probability of success when *ICT*_*ijk*_ and *X*_*ijk*_ change, *ceteris paribus*.

Equation (2) can be estimated by the conventional OLS method where the estimate of the coefficient of *ICT* indicates the impact of ICT innovation on food security. That is, $\hat{\gamma }$ measures the predicted change in the probability of success when *ICT*_*ijk*_ increases by one unit, *ceteris paribus*. This outcome potentially renders the interpretation meaningless as the ${\hat{\gamma }}_{j}$ may yield values outside 0 and 1 bound. Similarly, though OLS estimates remain unbiased but inefficient, *u*_*ijk*_ is not normally distributed but follows a binomial distribution. Equation (2) is also heteroskedastic because the variance of *u*_*ijk*_ depends on *P*_*ijk*_ which differs across every cross-sectional units. Hence, an alternative model that restricts the values of ${\hat{Y}}_{ijk}$ is required.

From Cassy, Natário, and Martins [40], when analyzing survey data where the interest is to predict a binary outcome from an explanatory variable and a set of covariates, the use of the logistic regression model is common. The regression model is in the class of limited dependent variable models for studying the relationship between a binary response variable *Y*, representing success (*Y* = 1) or failure (*Y* = 0) and a set of covariates *x* = (*x*_1_, *x*_2_, ⋯,*x*_*p*_)′. The set of covariates can be categorical or continuous or combinations of both. This study adopts the logistic model of Diaz-Perez et al. [38] and Hosmer, Lemeshow, and Sturdivant [41] and specifies a simplified generalized model given as

(5) $$\begin{array}{c} \pi \left(Y\right)=\frac{\exp\left(\alpha +\gamma {ICT}_{ijk}+\sum_{m=1}^{p}b_{m}X_{mijk}\right)}{1+\exp\left(\alpha +\gamma {ICT}_{ijk}+\sum_{m=1}^{p}b_{m}X_{mijk}\right)}=\frac{\exp^{\left(\alpha +\gamma {ICT}_{ijk}+\sum_{m=1}^{p}b_{m}X_{mijk}\right)}}{1+\exp^{\left(\alpha +\gamma {ICT}_{ijk}+\sum_{m=1}^{p}b_{m}X_{mijk}\right)}}. \end{array}$$

Equation (5) is estimated by maximum likelihood technique [42] and to verify that $\hat{\gamma }$ differs from 0; the Wald test shown in Equation (6) is used:

(6) $$\begin{array}{c} Z_{\text{Wald}}=\frac{\hat{\gamma }}{{\text{SE}}_{\hat{\gamma }}}. \end{array}$$

Such that $\hat{\gamma }$ is the estimate, and SE is the standard error of the regression. The analysis of *Z* allows a good interpretation of the regression, and the odds ratio [43] is written mathematically as

(7) $$\begin{array}{c} \theta =\frac{{\text{odds}}_{1}}{{\text{odds}}_{2}}=\frac{\pi \left(1\right)/1-\pi \left(1\right)}{\pi \left(2\right)/1-\pi \left(2\right)}, \end{array}$$

where the odds of an event occurring represent the ratio between the probability that the event will occur and the probability that the event will not occur, and *π*(*Y*) is the probability of a household having food security. In similar manner, the estimates of the coefficients of other covariates can be interpreted as the logarithms of the odds ratio.

## 3. Results and Discussion

### 3.1. Descriptive Statistics

Statistics shown in Table 3 reveal that out of 7,634 households, 3,713 (48.6%) are headed by males, and 3,921 (51.4%) are headed by females. Similarly, the percentage of households located in urban areas is 28.3% with 71.7% located in the rural areas representing 2,157 and 5,477 households, respectively. The mean value of food security per household is 0.345 with a standard deviation of 0.476. Likewise, on average, 0.684 households have access to a mobile phone with similar proportion for males and females. The average age in the data is 24.71 years with 23.55years for males and 25.86 years for females.

### 3.2. Econometric Result

Based on the study's objectives, the findings are presented in Table 4. The findings show that among the male household, female household, and total household ICT utilization, only the male household ICT utilization significantly and positively contributes to food security in Nigeria. This finding is unique because it provides evidence that activities, which positively drive food security that is mostly carried out by the male households, and furthermore, the male household relatively utilizes household ICT productively. Specifically, the findings show that a 1 percent increase in household ICT utilization among males significantly impacts food security by 0.68 percent. This significant finding is in line with a study by Issahaku, Abu, and Nkegbe [44], which used the PSM model to prove that ICT-mobile phone usage significantly improves agricultural productivity in Ghana, another West African country. Specifically, according to Issahaku et al. [44], ICT-mobile phone usage enhances household yields by at least 261.20 kg/ha per output season, which resulted to food security.

However, total household ICT utilization and female household utilization were found to be insignificant and negative, respectively, in explaining the level of food security in Nigeria. This outcome is not surprising and not unrelated to the fact that, first, most farmers in households are the male farmers; hence, the females are not interested in the farming activities as such activities are the responsibilities of the men. Second, most farmers' mobile devices are not sophisticated enough to perform advanced functions such as online transactions that are significantly helpful in driving food security. Third, majority of farmers (about 80%) lack access to the internet, and less than 5% of households receive E-wallet fertilizer and improved seedling information which could significantly improve productivity and contribute to food security in Nigeria. To further buttress the discussion, Figures 1, 2, and 3 in the supplementary documents show the proportion of ICT adopted by household. The graphical depiction shows that though households' own phones are without internet access as a result, there is little food access via that medium to, for example, order farm implements and perform other transactions that could aid productivity and in turn, food security.

With regard to the other variables, agricultural productivity is statistically significant at the 5% level in explaining food security in all households, male headed households, and urban area households. From the result, a 1% increase in agricultural productivity is capable of increasing food security by approximately 1.44% (all household), 2.01% (male household), and 2.20% (urban households). This result aligns with “*apriori*” expectations. Though, agricultural productivity is a requisite for food security, however, coupled with the lack of ICT among rural households and the use of crude implements and labor-intensive farming mechanism that brings about low yields. This finding is akin to Osabohien et al. [10] who posits that households' access to ICT, and credit facilities are necessary ingredients for productivity among rural households [45, 46]. Their results reveal that households with access to credit and other farm implements had yields thrice more than households without access to credit.

Similarly, labor wage and labor hour are significant in explaining the level of food security among Nigerian households. With respect to labor hour, among all households in the sample and male headed households, labor hour is not statistically significant, but statistically significant among female headed and urban households. This implies that a 1% increase in labor hour leads to an increase in food security by 0.88% (female household) and by 0.93% (urban households), respectively. Labor wage shows to increase food security by 0.62% (all households), 0.87% (female households), and 0.82% (rural households), respectively. Labor wage may increase or reduce food security, depending on the ability of households to manage hired labor. This is because, as households pay between N500 to N2000 (currency denoted in Nigerian Naira) per day for a hired labor, increase in labor wage may lower the ability of households to hire more labor required for the cultivation of a specific plot of land; however, the ability of households to hire more labor may increase harvest. Labor hours (that is, hours spent in agriculture) may increase or have no impact on food security [27, 41].

Among households' characteristics, educational qualification and state of origin, age marital status, and enumeration areas are significant in explaining food security among households. The estimated coefficients of the robustness check presented in Table 5 are somewhat similar to the estimated coefficient of the main regression, but with little difference in the significance levels.

## 4. Conclusions

Aligning with goal 2 of the 2030 Sustainable Development Goals, to “end hunger, achieve food security, improved nutrition, and promote sustainable agriculture,” this study is motivated by the apparent gap in literature and the need to investigate the household ICT utilization and food security nexus in Nigeria. Using the World Bank LSMS (wave 4, 2018/2019) survey data and the logit regression technique, findings reveal that ICT utilization by male farming households in Nigeria plays a statistically significant role on food security, while for the female households, an insignificant and, however, negative nexus is observed with food security in Nigeria. Furthermore, the findings show that for male household users, a 1 percent increase in male household ICT utilization spurs about 0.68 percent increase in food security in Nigeria. The findings imply that among the male and female household ICT users, the male household ICT utilization significantly contributes to food security in Nigeria. Further scrutiny of the household data showed that first, although most of the households in the survey have access to mobile phones, these devices are not sophisticated and also connected to the internet which reduces the ability for the household to harness the benefits of ICT utilization on food security in Nigeria.

Policy recommendations are not far-fetched. Based on the findings of the study, it is expedient that measures be taken by relevant stakeholders to ensure that the potentials of ICT be fully maximized by farming households to contribute to food security in the nearest future. As suggestions for future studies, the literature can be improved upon by establishing linkages between household technology utilization shocks and food security in Nigeria using empirical techniques of estimation including the vector autoregressive (VAR) and the structural VAR along with the related impulse response functions and variance decompositions. Furthermore, a panel study on Africa could be carried out to further test the ICT-food security hypothesis on a wider scope-Africa, in addition to cross-sectional and comparative studies being carried out on the theme of this study for a robust finding.

## Figures and Tables

**Table 1:** Variable description and measurement.

Variables	Description	Measurement
Food security status	Measures the food security status of the household	=1 if food secured and 0 otherwise
Mobile phone utilization	Measure of ICT	
Labor hour	Hours worked per day on the farm	Natural logarithm of hours worked
Labor wage	Wages paid by households to labor	Natural logarithm of wages paid by households
Educational qualification	Highest qualification attained	Polychotomous variablee (see Table 2 for details)
Age	Age in completed years	Years
Marital status	Marital status of household head	Polychotomous variable (see Table 2 for details)
State of origin	State of origin of the household	Polychotomous variable (see Table 2 for details)
Enumeration area	Location of household	1 = urban, 2 = rural
Sector	Area study is conducted	
Sex	Sex of household head	1 = male, 2 = female
Assistance	Support received by households	Polychotomous variable (see Table 2 for details)

Source: authors' compilation.

**Table 2:** Variables and measurement.

Variable	Composition
Educational qualification	None, first school leaving certificate, modern school leaving certificate, junior secondary school certificate, senior secondary school certificate, advanced (A) level, NCE/OND/nursing, BA/BSc/HND, PhD/masters, doctorate, other (specify), Voc/Comm certificate, Voc/Comm diploma
Marital status	Married (monogamous), married (polygamous), informal union, divorced, separated, widowed, never married
State of origin	Abia, Adamawa, Akwa Ibom, Anambra, Bauchi, Bayelsa, Benue, Borno, Cross River, Delta, Ebonyi, Edo, Ekiti, Enugu, Gombe, Imo, Jigawa, Kaduna, Kano, Katsina, Kebbi, Kogi, Kwara, Lagos, Nasarawa, Niger, Ogun, Ondo, Osun, Oyo, Plateau, Rivers, Sokoto, Taraba, Yobe, Zamfara, FCT

Assistance	Free food/maize distribution, food/cash-for-work program, inputs-for-work program, targeted nutrition program, and direct cash transfer from govt.

Source: authors.

**Table 3:** Summary statistics.

Variables	Full sample		Household by sex				Household by sector			
			Male		Female		Urban		Rural	
	Mean	Std. Dev.	Mean	Std. Dev.	Mean	Std. Dev.	Mean	Std. Dev.	Mean	Std. Dev.
Food security status	.34	.47	.34	.47	.34	.47	.39	.48	.32	.46
Mobile phone utilization	.68	.072	.68	.06	.68	.07	.68	.07	.68	.071
Labor hours	1.68	.34	1.68	.33	1.68	.35	1.68	.34	1.68	.34
Labor wages	6.97	.52	6.97	.50	6.98	.55	7.03	.55	6.96	.51
Educational qualification	3.64	4.13	3.62	4.09	3.67	4.17	3.71	4.14	3.62	4.12
Age	24.71	20.02	23.5	19.87	25.85	20.09	24.76	20.30	24.69	19.90
Marital status	5.27	2.57	5.46	2.54	5.09	2.59	5.31	2.55	5.26	2.57
State of origin	14.25	9.40	14.11	9.34	14.37	9.46	16.33	9.62	13.43	9.19
Enumeration area	898.32	827.90	906.53	851.73	890.54	804.72	893.60	802.93	900.18	837.60
Sector	1.71	.45	1.71	.44	1.71	.45				
Sex	1.51	.49					1.51	.49	1.51	.49
Assistance	.64	.17	.64	.17	.64	.18	.62	.20	.64	.16
Observations	7634		3713		3921		2157		5477	

Source: authors' compilation.

**Table 4:** Logit regression estimates (outcome variable: food security).

Variable	All households	Male households	Female households	Urban households	Rural households
Constant	-1.0611	-0.9120	0.5837	-3.3638	-3.4034
	(2.4039)	(3.4796)	(6.7399)	(3.0181)	(2.7563)
	[0.659]	[0.7930]	[0.9310]	[0.2650]	[0.2170]

Productivity	1.4386^∗∗^	2.009^∗∗^	0.8645	2.1954^∗∗^	1.0286
	(0.5722)	(0.7793)	(0.9113)	(1.0488)	(0.6651)
	[0.012]	[0.0100]	[0.3430]	[0.0360]	[0.1220]

Labor hour	0.0806	-0.7981	0.8804^∗∗∗^	0.9283^∗∗∗^	0.0941
	(0.3370)	(0.5249)	(0.4895)	(0.5437)	(0.4046)
	[0.8110]	[0.1280]	[0.0720]	[0.0880]	[0.8160]

Labor wage	0.6236^∗∗^	0.4016	0.8683^∗∗^	0.2224	0.8172^∗^
	(0.2428)	(0.3595)	(0.3559)	(0.3686)	(0.3067)
	[0.0100]	[0.2640]	[0.0150]	[0.5460]	[0.0080]

ICT	-2.958607	0.6828	-10.3423		-2.414905
	(1.9419)	(2.6761)	(9.0702)		(1.9976)
	[0.1280]	[0.7990]	[0.2540]		[0.2270]

Assistance	0.4248	1.226882	0.1008	-0.7311	0.3966
	(0.5967)	(0.9478)	(0.8965)	(1.2308)	(0.6428)
	[0.4770]	[0.1960]	[0.9110]	[0.5520]	[0.5370]

Household characteristics					
Educational qualification	0.0803^∗∗^	0.0812	0.0999^∗∗∗^	0.0223	0.0805^∗∗^
	(0.0343)	(0.0560)	(0.0515)	(0.0456)	(0.0399)
	[0.0190]	[0.1470]	[0.0520]	[0.6250]	[0.0430]

State of origin	-0.0269^∗∗^	-0.0140	-0.0450^∗∗^	-0.0423^∗∗∗^	-0.0260^∗∗∗^
	(0.0114]	(0.0161)	(0.0173)	(0.0220)	(0.0132)
	[0.0190]	[0.3840]	[0.0090]	[0.0540]	[0.0490]

Sector	-0.5789^∗∗^	-0.7721^∗∗^	-0.5049		
	(0.2641)	(0.3872)	(0.3975)		
	[0.0280]	[0.0460]	[0.2040]		

Age	-0.0153^∗∗^	-0.0223^∗∗∗^	-0.0142	0.0117	-0.0211^∗∗^
	(0.0071)	(0.0122)	(0.0094)	(0.0117)	(0.0086)
	[0.031]	[0.0680]	[0.1320]	[0.3180]	[0.0140]

Sex	-0.3155			-0.4898	-0.2562
	(0.222]			(0.4058)	(0.2562)
	[0.1560]			[0.2270]	[0.3170]

Marital status	-0.0972^∗∗∗^	-0.1486	-0.0678	0.1111	-0.1450
	(0.0542)	(0.0920)	(0.0744)	(0.0962)	(0.0636)
	[0.0730]	[0.1060]	[0.3620]	[0.2480]	[0.0230]

Enumeration Area	-0.0004^∗∗^	-0.0005^∗∗^	-0.0003	-0.0002	-0.0004^∗∗∗^
	(0.0002)	(0.0002)	(0.0003)	(0.0003)	(0.0002)
	[0.0230]	[0.0330]	[0.2550]	[0.5070]	[0.0480]

Log likelihood	-238.1668	-119.2972	-110.8883	-73.9609	-181.6230
observation	377	190	187	116	289
Probability	0.0000	0.0109	0.0003	0.0799	0.0007
Pseudo-R2	0.0841	0.0930	0.1350	0.2320	0.0812

Note: ^∗^, ^∗∗^, and ^∗∗∗^ represent statistical significance at 1%, 5%, and 10% levels, respectively. Standard errors and *p* values are in the parentheses () and , respectively. Source: authors' computations.

**Table 5:** Robustness estimates (outcome variable: food security).

Variable	All households	Male household	Female household	Urban household	Rural households
Constant	-1.0611	-0.9120	0.5837	-3.3638	-3.4033
	(2.6097)	(3.4872)	(6.1222)	(3.2302)	(2.8832)
	[0.684]	[0.7940]	[0.9240]	[0.2980]	[0.2380]

Productivity	1.4386^∗∗^	2.0087	0.8645	2.1954	1.02856
	(0.6045)	(0.8570)	(0.8835)	(1.1261)	(0.6885)
	[0.0170]	[0.0190]	[0.3280]	[0.0510]	[0.1350]

Labor hour	0.0806	-0.7981	0.8804^∗∗∗^	0.9283^∗∗∗^	0.0941
	(0.3398)	(0.5800)	(0.4486)	(0.5203)	(0.4051)
	[0.8120]	[0.1690]	[0.0500]	[0.0740]	[0.8160]

Labor wage	0.6236^∗∗^	0.4017	0.8683^∗∗^	0.2224	0.8172^∗^
	(0.2433)	(0.3485)	(0.3933)	(0.3640)	(0.3071)
	[0.0100]	[0.2490]	[0.0270]	[0.5410]	[0.0080]

ICT	-2.9587	0.6828	-10.3423		-2.4149
	(2.4082)	(2.4774)	(7.7746)		(2.4205)
	[0.2190]	[0.7830]	[0.1830]		[0.3180]

Safety nets/assistance	0.4248	1.2269	0.1008	-0.7311	0.3966
	(0.6089)	(0.9983)	(0.8579) [0.907]	(1.2252)	(0.6279)
	(0.4850]	[0.2190]		[0.5510]	[0.5280]

Household characteristics					
Educational qualification	0.0803^∗∗^	0.0812	0.0999 ^∗∗^	-0.0223	0.0805^∗∗^
	(0.0289)	(0.0566)	(0.0399)	(0.0389]	(0.0327)
	[0.0050]	[0.1520]	[0.0120]	[0.5670]	[0.0140]

State	-0.0269^∗∗^	-0.0140	-0.0451^∗∗^	-0.0424^∗∗∗^	-0.0260^∗∗^
	(0.0115)	(0.0154)	(0.0174)	(0.0233)	(0.0130)
	[0.0190]	[0.3640]	[0.0100]	[0.0690]	[0.0470]

Sector	-0.5789^∗∗^	-0.0140	-0.5049		
	(0.2636)	(0.0154)	(0.3961)		
	[0.0280]	[0.3640]	[0.2020]		

Age	-0.0153^∗∗^	-0.0223^∗∗^	-0.0142 (0.0107)	0.0117	-0.0211
	(0.0073	(0.0114)	[0.1850]	(0.0117)	(0.0086)
	[0.0370]	[0.0500]		[0.317]	[0.0140]

Sex/gender	-0.3155			-0.4898	-0.2562
	(0.2235)			(0.4171)	(0.25679)
	[0.1580]			[0.2400]	[0.3180]

Marital status	-0.0972^∗∗∗^	-0.1486^∗∗∗^	-0.0678	0.1110	-0.1450^∗∗^
	(0.0543)	(0.0884)	(0.0732)	(0.0975)	(0.0621)
	[0.0740]	[0.0930]	[0.3540]	[0.2550]	[0.0200]

Enumeration area	0.0803^∗∗^	-.0005^∗∗^	-0.0003	-0.0002	-0.0004^∗∗^
	(0.0289)	(0.0002)	(0.0003)	(0.0003)	(0.0002)
	[0.047]	(0.0290]	[0.2470]	[0.5280]	[0.0390]

Log likelihood	-238.1668	-119.2971	-110.88831	-73.9609	-181.6230
Observation	377	190	187	116	289
Probability	0.0000	0.0431	0.0067	0.3138	0.0021
Pseudo-R2	0.0841	0.0930	0.1350	0.0799	0.0812

Note: ^∗^, ^∗∗^, and ^∗∗∗^ represent statistical significance at 1%, 5%, and 10%, respectively; standard errors and *p* values are in the parentheses () and , respectively. Source: authors' computations.

## Data Availability

The data used for this research is available online at the World Bank database.

## Supplementary Materials

Supplementary Materials Figure 1: number of households with phones. Figure 2: number of households with access to the Internet. Figure 3: households who received E-wallet fertilizer and improved seed information.
