# Supplementary material for: Household ICT Utilization and Food Security Nexus in Nigeria
Source: Int J Food Sci. 2021 Nov 10;2021:5551363. doi: 10.1155/2021/5551363 (PMC8598372; doi:10.1155/2021/5551363)
Supplement: Supplementary Materials — Figure 1: number of households with phones. Figure 2: number of households with access to the Internet. Figure 3: households who received E-wallet fertilizer and improved seed information. [file 5551363.f1.docx]

**Figure 1: Number of Households with Phones**

c.

b.

A

**Figure 2: Number of Households with Access to the Internet**

c.

b.

A

**Figure 3: Households who Received E-wallet Fertilizer and Improved Seed Information**

c.

b.

A
